# Seroprevalence of *Helicobacter pylori* infection and its related risk factors in symptomatic patients in southern Ethiopia

**DOI:** 10.1186/1756-0500-7-834

**Published:** 2014-11-24

**Authors:** Endale Tadesse, Deresse Daka, Demo Yemane, Techalew Shimelis

**Affiliations:** Department of Medical Laboratory Science, College of Medicine and Health Sciences, Hawassa University, P.O. Box 1560, Hawassa, Ethiopia

**Keywords:** *Helicobacter pylori*, ABO blood groups, Seroprevalence

## Abstract

**Background:**

*Helicobacter pylori* is the main etiology of peptic ulcers and chronic gastritis. Various studies showed that blood type ‘O’ is more common among patients with peptic ulcer. The aim of this study was to determine the seroprevalence of *H. pylori* antibodies and its relationship with ABO/Rhesus blood groups, age, sex and residence of symptomatic patients in southern Ethiopia.

**Methods:**

A cross-sectional study was conducted in a total of 408 consecutive patients with upper abdominal complaints at Hawassa University Hospital from October 2012 to January 2013. Data on demographic factors was collected from all participants using questionnaires. Blood samples were also collected and tested for ABO and Rh blood group phenotype using hemagglutination test and for anti-*H. pylori* antibody (IgG) using two different ELISAs..

**Results:**

The overall seroprevalence of *H. pylori* infection was 83.3% (340/408), and it was significantly higher in rural (71.2%) compared to urban residents (28.8%) (p = 0.008). Participants with blood group AB, A, O, B, and Rh positive had *H. pylori* prevalence of 88.9, 84.2, 83.7, 80.9, and 83.5%, respectively. *H. pylori* infection was not significantly influenced by age, sex, occupation, educational status and ABO/ Rh status (p >0.05).

**Conclusion:**

The high seroprevalence of *H. pylori* infection especially among rural residents calls for immediate intervention measures so that its clinical consequences could be minimized. ABO/Rh blood group was not found to be associated with *H. pylori* infection.

## Background

*Helicobacter pylori* is a small, spiral-shape, Gram-negative bacillus that inhabit the mucous layer overlying the gastric epithelial cells in humans [1]. The organism has been etiologically associated with chronic active gastritis [2], peptic ulcer disease [3], gastric cancer [4] and mucosal associated lymphoid tissue (MALT) lymphoma [5]. It is estimated that up to 50% of the world population are infected with *H. pylori* [6]. Prevalence is higher in developing countries than developed nations [7], and varying within and among countries [8]. In Ethiopia, studies have showed the high prevalence of *H. pylori* infection among adults in various localities [9, 10], similar to results from other developing countries [11, 12]. As *H. pylori* is largely transmitted by the fecal-oral route, poor hygiene practice, overcrowding and lack of sanitation are important factors for its preponderance in developing countries [8]. An experience of developed nations showed that improvement of hygiene condition significantly decreased the prevalence of the infection [13].

Despite high prevalence of *H. pylori* infection, most infected people remain asymptomatic and only minorities develop peptic ulcer disease [11]. It may be the host genetic factors and/or *H. pylori* strains that determine the clinical significance of the infection. Boren *et al.* reported that individuals with blood group O and with Lewis b antigen more likely develop gastritis, since these antigens mediate the attachment of *H. pylori* to the gastric mucosa [14]. It was also shown that the frequency of O blood group and non-secretor phenotype of ABO antigens are higher among patients with peptic ulcers [15–19]. However, several studies reported absence of association between *H. pylori* infection and ABO blood groups [20–22].

In developing nations, the required health finance to properly manage *H. pylori* infection, which results in diseases including peptic ulcer and gastric cancer, is unaffordable [11]. Thus, planning prevention measures that reduce the public health significance of *H. pylori* infection is critically needed. In this regard, investigating the epidemiology of the infection in various localities is required to design effective intervention measures. Therefore, this study aimed at assessing the seroprevalence of *H. pylori* infection and its related risk factors in southern Ethiopia.

## Methods

### Study area and design

A cross-sectional study was conducted at Hawassa Teaching and Referral Hospital, in southern Ethiopia, from October 2012 to January 2013. The hospital is situated in Hawassa, the capital city of the Southern Nations, Nationalities and People’s Regional state in Ethiopia, and the largest public hospital in the administrative region. Patients with clinical indications for *H. pylori* infection are routinely tested in the hospital using rapid serological diagnostic tests.

### Study population and sample size

The study population consisted of patients examined at the outpatient department of the Hospital and with clinical indications of *H. pylori* infection. Participants less than 15 years of age and who took treatment against *H. pylori* infection a month prior to this study were excluded. In total, 408 consecutive patients were included in the study.

### Data collection

A trained nurse collected data on socio-demographic factors such as age, sex, residence, occupation, and educational level using questionnaires. About 5 ml of venous blood were collected from the participants for blood grouping and serology. Blood samples were allowed to clot and centrifuged at 3000 RPM for 10 minutes; sera were separated and refrigerated at -20°C until tested.

### Blood grouping

ABO/Rh blood grouping was performed using direct tube hemagglutination method.

### Serology

Sera were tested for anti-*H. pylori* immunoglobulin G (IgG) using two different enzyme linked immunosorbent assay (ELISA) kits: Pyloriset EIA-G III (Orion Diagnostica, Germany) and *H. pylori* IgG ELISA (IBL International, Hamburg, Germany). The procedure for the ELISAs was described elsewhere [23], and concordant results determined *H. pylori* infection status of the participants.

### Data analysis

Data entry and analysis was performed using SPSS Version-16. Results were summarized using descriptive statistics. Pearson’s Chi-square (*X*^2^) test was used to evaluate differences between proportions and p-value less than 0.05 was considered statistically significant.

### Ethical consideration

The study was approved by the Institutional Review Board of College of Medicine and Health Sciences, Hawassa University. Participation was fully voluntary and informed consent was obtained from each study participant. Screening for *H. pylori* infection was performed free of charge, and those found positive was managed by physicians.

## Results

### Study participants

Out of 419 patients approached during the study period, 11 (2.6%) were excluded because of 3 patients were on anti-*H. pylori* treatment, 1 refused to participate, 4 had discordant ELISA results and 3 were children under 15 years old. Thus, data from 408 patients was considered for analysis. Participants had mean age of 35 years (range; 15 – 96 years) and the male to female ratio was 1.04:1. The mean age of the study subjects was 35 years (range, 15 – 96 years), and majorities, 305 (74.8%), were below the age of 45 years. The majority of the participants (73.6%) were rural residents and farmers (20.6%) by occupation. Blood group O was the most frequent (40.7%) phenotype followed by type A (27.9%), type B (27.0%), and type AB (4.4%). The vast majority of the participants were Rh positive (96.8%).

### *H. pylori*infection and its distribution by socio-demographic factors

**Figure 1:**
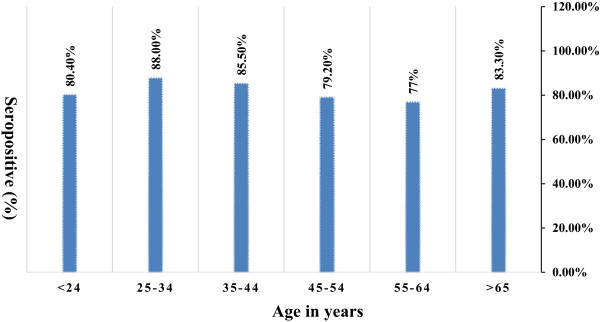
**Seropositivity of - infection among different age group in southern Ethiopia, 2012-2013.**

The overall prevalence of *H. pylori* infection was found to be 83.3% (340/408) by double ELISA testing. The distribution of infection by socio demographic factors is presented in Table 1. The infection occurred more frequently among male participants (51.8%) compared to females (48.2%) though the difference was not statistically significant (p = 0.508). As shown in Figure 1, exposure to *H. pylori* seems to be higher among participants in the age group 25 – 34 years (88%) though age was not found to be associated with infection status (p = 0.479). However, the infection occurred in significantly higher rate among residents in rural compared to urban sites (71.2 versus 28.8%; p = 0.008).

**Table 1:** **Socio-demographic characteristics and seropositivity of** ***H. pylori*** **infection in southern Ethiopia, 2012-2013**

Characteristics	Total tested No (%)	Seropositive N = 340	Seronegative N = 68	***X*** ^2^-value	p-value
		No (%)	No (%)		
Sex (M/F)				0.502	0.508
M	208 (51)	176 (51.8)	32 (47.1)		
F	200 (49)	164 (48.2)	36 (52.9)		
Occupation				4.396	0.820
Farmer	84 (20.6)	71 (84.5)	13 (15.5)		
House wife	79 (19.4)	64 (81)	15 (19)		
Gov’t employee	70 (17.2)	58 (82.9)	12 (17.1)		
NGO Employee	58 (14.2)	46 (79.3)	12 (20.7)		
Student	71 (17.3)	66 (93)	5 (7)		
Merchant	26 (6.4)	21 (80.8)	5 (19.2)		
Un employee	20 (4.9)	14 (70)	6 (30)		
Residency				7.117	0.008
Rural	301 (73.8)	242 (71.2)	59 (86.8)		
Urban	107 (26.2)	98 (28.8)	9 (13.2)		
Educational statues				0.882	0.348
Illiterate	136 (33.3)	110 (80.9)	26 (19.1)		
literate	272 (66.7)	230 (84.6)	42 (13.4)		
Total	408 (100)	340 (83.3)	68 (16.7)

The distribution of *H. pylori* infection among participants with different blood types indicated that those with blood type AB, A, O, and B had 88.9, 84.2, 83.7 and 80.9% rate of infection, respectively (Table 2). RH positive individuals had higher rate of exposure to *H. pylori* compared to RH negatives (83.5% versus 76.9%). However, neither ABO blood type (p = 0.814) nor RH status (p = 0.535) significantly influenced *H. pylori* infection.

**Table 2:** **Association of ABO/Rh blood group and** ***H. pylori*** **infection in southern Ethiopia, 2012-2013**

Blood group	Total tested	Seropositive (n = 340)	Seronegative (n = 68)	***X*** ^2^ -value	p- value
	No (%)	No (%)	No (%)		
ABO blood group (n = 408)				0.948	0.814
O	166 (40.7)	139 (83.7)	27 (16.3)		
A	114 (27.9)	96 (84.2)	18 (13.8)		
B	110 (27)	89 (80.9)	21 (19.1)		
AB	18 (4.4)	16 (88.9)	2 (11.1)		
Rh blood group (n = 408)				0.386	0.535
Rh negative	395 (96.8)	10 (76.9)	3 (23.1)		
Rh positive	13 (3.2)	330 (83.5)	65 (16.5.6)		

## Discussion

Overall, the seroprevalence of *H. pylori* infection among symptomatic patients attending the outpatient department of Hawassa Teaching and Referral Hospital was 83.3%, which is in agreement with results reported from Ethiopia; 85.6% in Gondar [10] and 69 – 91% in Addis Ababa [9, 24]. In contrast, our finding was higher than other results reported from Ethiopia; 49 – 70% in Bahirdar [20], 56 – 70%) in Addis Ababa [25, 26] and 66% in Gondar [27]. Also, the present result was found to be lower than the figures reported in Iran (46.6 – 64.8%) [18, 28, 29], Turkey (68%) [21], and in Portugal City of Coimbra (67%) [19]. The higher prevalence of *H. pylori* infection may be related to poor personal hygiene, low standard of living and low economic status among dyspeptic patients in the study area.

Although the significance of age to influence *H. pylori* infection was shown elsewhere [7, 10, 21, 17, 27], this contrasts our and others’ findings [20, 26, 28]. Moreover, the role of sex to differentially put men at higher risk of infection compered to women was also shown by others [17, 30, 31] though this could not be revealed in the current and similar previous studies [9, 20, 21, 27, 28]. Our finding, which indicated the higher prevalence of *H. pylori* infection in rural residents compared to urban, was in line with a report elsewhere [21], and it may be attributed to factors related to the lack of safe water supply and hygiene condition in the rural part of the country.

As to the relation between ABO blood group and *H. pylori* infection, contrasting evidences exists in which some studies revealed the presence of association [17–19], while others not [2, 20–22, 24, 29]. Similar to the latter groups, we observed neither ABO nor Rh was significantly associated with infection in our study population, which challenges reports indicating participants with blood type O are more prone to *H. pylori* infection.

*H. pylori* infection was detected using serological test, which is acceptable for epidemiological studies. However, serological tests unable to differentiate current infection from previous infections. Thus, in order to reveal whether the finding reported in this study reflects the actual situation of *H. pylori* infection in the general population, further community based studies should be conducted using different diagnostic methods such as culture, histological test, urease test, serology and gram staining.

## Conclusions

In conclusion, the seroprevalence of *H. pylori* infection was high among symptomatic patients at Hawassa University Hospital. Though the infection status was significantly affected by residence site, no association was observed with age, sex, occupation, educational status and ABO/Rh blood type. The burden of *H. pylori* that we reported warrants the need to design and implement intervention measures that could reduce transmission, and thus lessen the clinical consequences of infection.
